# Development and Pragmatic Randomized Controlled Trial of Healthy Ketogenic Diet Versus Energy-Restricted Diet on Weight Loss in Adults with Obesity

**DOI:** 10.3390/nu16244380

**Published:** 2024-12-19

**Authors:** Su Lin Lim, Melissa Tay, Siew Min Ang, Shu Ning Wai, Kai Wen Ong, Wen Joo Neo, Qai Ven Yap, Yiong Huak Chan, Chin Meng Khoo

**Affiliations:** 1 Office of Allied Health & Pharmacy, National University Hospital, Singapore 119228, Singapore; 2 Department of Dietetics, National University Hospital, Singapore 119074, Singapore; melissa_hj_tay@nuhs.edu.sg (M.T.); siewmin1912@gmail.com (S.M.A.); whyshuning@gmail.com (S.N.W.); dtn.kaiwen@gmail.com (K.W.O.); wen_joo_neo@nuhs.edu.sg (W.J.N.); 3 Biostatistics Unit, Yong Loo Lin School of Medicine, National University Singapore, Singapore 117597, Singapore; qaiven@nus.edu.sg (Q.V.Y.); yionghuak@singnet.com.sg (Y.H.C.); 4 Department of Medicine, National University Hospital, Singapore 119074, Singapore; chin_meng_khoo@nuhs.edu.sg

**Keywords:** healthy ketogenic diet, energy-restricted diet, weight loss, obesity, metabolic outcomes, Asian, adults

## Abstract

**Introduction**: The ketogenic diet (KD) is widely used for weight management by reducing appetite, enhancing fat oxidation, and facilitating weight loss. However, the high content of total and saturated fats in a conventional KD may elevate low-density lipoprotein (LDL)-cholesterol levels, a known risk factor for cardiovascular diseases, highlighting the need for healthier alternatives. This study aimed to investigate the effect of a newly developed Healthy Ketogenic Diet (HKD) versus an Energy-Restricted Diet (ERD) on weight loss and metabolic outcomes among adults with obesity. **Methods**: Multi-ethnic Asian adults (*n* = 80) with body mass index ≥ 27.5 kg/m^2^ were randomized either to HKD (*n* = 41) or ERD *(n* = 39) for 6 months. Both groups followed an energy-restricted healthy diet, emphasizing on reducing saturated and trans fats. The HKD group additionally limited net carbohydrate intake to no more than 50 g per day. Dietary adherence was supported via the Nutritionist Buddy app with dietitian coaching. The primary outcome was weight change from baseline at 6 months. Secondary outcomes included weight change at 3 months and 1 year, along with changes in metabolic profiles. Data were analyzed using linear regression with an intention-to-treat approach. **Results**: The HKD group achieved significantly greater mean weight loss at 6 months than the ERD group (−7.8 ± 5.2 kg vs. −4.2 ± 5.6 kg, *p* = 0.01). The mean weight loss percentage at 6 months was 9.3 ± 5.9% and 4.9 ± 5.8% for the HKD and ERD groups, respectively (*p* = 0.004). Improvements in metabolic profiles were also significantly better in the HKD group [glycated hemoglobin (−0.3 ± 0.3% vs. −0.1 ± 0.2%, *p* = 0.008), systolic blood pressure (−7.7 ± 8.9 mmHg vs. −2.6 ± 12.2 mmHg, *p* = 0.005), and aspartate transaminase (−7.6 ± 15.5 IU/L vs. 0.6 ± 11.5 IU/L, *p* = 0.01)], with no increase in LDL-cholesterol (−0.12 ± 0.60 mmol/L vs. −0.04 ± 0.56 mmol/L, *p* = 0.97) observed in either group. **Conclusions**: The HKD was more effective than the ERD in promoting weight loss and improving cardiometabolic outcomes without elevation in LDL-cholesterol. It can be recommended for therapeutic intervention in patients with obesity.

## 1. Introduction

The ketogenic diet (KD) comprising a low-carbohydrate, high-fat intake has been recognized for its potential benefits in weight loss [1]. It facilitates weight loss by reducing appetite, increasing satiety, enhancing fat burning through ketosis, and improving insulin sensitivity [2,3,4]. The traditional ketogenic dietary approach restricts net carbohydrate intake to below 50 g per day, prioritizing non-starchy vegetables while excluding sugary and starchy foods, yet allowing for a high intake of fats regardless of whether they are considered healthy or unhealthy fats [5]. Despite its widespread adoption, the high intake of total fat (up to 90% of energy intake) and saturated fats has been associated with higher levels of low-density lipoprotein (LDL)-cholesterol, which is a well-established risk factor of cardiovascular disease (CVD) [2,6,7]. Unlike many other weight loss approaches, the ketogenic diet does not restrict energy intake, which may seem counter intuitive for weight maintenance following successful weight loss [8,9].

Furthermore, ketogenic diets frequently lack sufficient fiber, a crucial element for promoting satiety and insulin sensitivity. Satiety and insulin sensitivity, in turn, play a pivotal role in enhancing weight loss and cardiovascular health outcomes [10,11]. On the gastrointestinal health front, a high-fiber diet fosters the generation of beneficial colonic short-chain fatty acids by gut microbiota and prevents constipation [12]. Therefore, incorporating high fiber and non-digestible carbohydrates into ketogenic diets may enhance nutrient intake and provide gastrointestinal benefits.

This study aimed to investigate the effect of HKD compared to the standard care of an ERD on weight loss and metabolic outcomes among individuals with obesity.

## 2. Materials and Methods

### 2.1. Study Design

This study was a pragmatic randomized controlled trial with open-label parallel arm assignment. The study was approved by the National Healthcare Group Domain Specific Review Board in Singapore (DSRB Ref: 2021/00833) and prospectively registered on ClinicalTrials.gov (Identifier: NCT0504995). All participants provided written consent prior to study participation.

### 2.2. Study Participants

Participants were public healthcare staff recruited from the National University Hospital in Singapore through email publicity broadcasts conducted between November 2021 and January 2023. The inclusion criteria were individuals aged 21 to 65 years with obesity (body mass index (BMI) 27.5–40 kg/m^2^) [13], literacy in English, and access to a smartphone. Participants with cancer, eating disorders, heart failure, advanced kidney disease, type 1 diabetes, type 2 diabetes on insulin, severe cognitive or psychological disabilities, depression, hypothyroidism, thalassemia or blood disorders, or who were pregnant were excluded from the study.

### 2.3. Development of the Healthy Ketogenic Diet (HKD)

The Healthy Ketogenic Diet (HKD) was designed to optimize weight loss and metabolic benefits associated with a KD while mitigating a rise in LDL-cholesterol levels. The HKD comprised eight key components: (i) energy restriction tailored to the individual was calculated using the Schofield equation, adjusted with at least 500 kcal deficit daily to promote weight loss [14]; (ii) a 50 g net carbohydrate daily limit (total carbohydrate constituting 20–25% of energy intake) [2,3,15]; (iii) low in saturated and trans fat [16,17]; (iv) total fat within 50% of energy intake with an emphasis on healthy fats such as monounsaturated fat and omega-3 fatty acids [16,17]; (v) adequate protein (25–30% of energy intake; 1.0–1.2 g/kg body weight) [9,15]; (vi) adequate fiber (20–30 g/day) [18,19,20]; (vii) adequate fluid of at least 2 L a day [21,22]; and (viii) adequate micronutrient intake through supplementation [23].

The development of the HKD is grounded in the evidence-based literature. Tailored energy restriction ensures individuals achieve an energy balance deficit necessary for weight loss, while modifications to the macronutrients aim to induce nutritional ketosis [3,15]. In addition, the energy restriction also helps participants to acclimate to a controlled amount of food intake, facilitating weight maintenance in the future. The emphasis on unsaturated fats over saturated and trans fats intended to prevent increases in LDL-cholesterol levels [16,17]. Adequate but not excessive protein intake helps to preserve lean body mass [9] while preventing potential burden to the kidneys and gluconeogenesis from excess intake [24]. The inclusion of adequate fiber as a therapeutic requirement of the HKD targeted at increasing satiety, improving insulin sensitivity, and providing overall health benefits [18]. Emphasis on adequate fluid intake helps to mitigate dehydration-related side effects, which may enhance adherence to the diet [3,8,9]. Lastly, including a multivitamin-multimineral supplement can help address the micronutrient needs of individuals following the HKD [23].

### 2.4. Randomization and Masking

Eligible participants were randomized into either the Energy-Restricted Diet (ERD) group or the Healthy Ketogenic Diet (HKD) group in a 1:1 allocation ratio. RStudio version 3.6.3. was used to generate randomization codes using blocks of 4, stratified by gender and BMI category (<35 and ≥35 kg/m^2^). Stratification of BMI into categories of less than or greater than 35 kg/m^2^ is essential as individuals with a BMI greater than 35 kg/m^2^ are classified as Class III obesity, which is associated with a higher risk of various chronic health conditions. By stratifying participants based on this threshold, we ensured a more balanced distribution of participants into the control and intervention groups which helps to control confounding variables related to obesity. A third party who was not involved in the study conducted the allocation using stratified sequentially numbered opaque envelopes. The envelopes were opened sequentially by the investigator after the participants had consented to participate, and the treatment group was assigned accordingly.

### 2.5. Intervention

All participants attended seven group workshops over the intervention period. Workshops were conducted by a dietitian and lasted around an hour each. The workshops covered nutrition content which comprised: (i) foundation of the diet assigned; (ii) utilizing technology and physical activities; (iii) eating out; (iv) home cooking; (v) supermarket tour and food labelling; (vi) breaking a weight plateau; and (vii) behavior change and tips for weight maintenance and diet recommendations post-intervention. All participants were advised to follow an energy-restricted diet emphasizing a reduction in saturated and trans fats. Additionally, participants in the HKD group were advised to adhere to a maximum of 50 g net carbohydrate intake daily.

Participants were given a digital weighing scale (Omron HN-289, Omron Healthcare Co. Ltd., Kyoto, Japan) to track their weight. They were instructed to use the Nutritionist Buddy (nBuddy) (Heartvoice Pte Ltd., Singapore) mobile app [25,26,27] to record their weight twice a week as well as their diet and physical activity daily. Regular communication with the dietitians on the app platform was also emphasized. Adherence to their tailored daily energy limit was recommended along with regular physical activity. Participants were advised to achieve their incremental daily step count goal from 3000 (1st week), to 7000 (2nd week), and to 10,000 (3rd week onwards) as tolerated. The dietitian checked in on patients and provided coaching via the chat function of the nBuddy app throughout the 6-month intervention period. Individualized feedback along with motivational techniques were offered based on the participants’ inputs on the app. This helped to guide participants towards making healthy lifestyle changes through timely feedback, barrier identification, and problem-solving. Educational videos on the diet assigned, exercise, and habit-building were also made available to the participants. After the 6-month program, no further intervention was provided. However, participants were allowed to continue using the nBuddy app without further coaching support.

### 2.6. Outcome Evaluation

The primary outcome was mean weight change from the baseline at 6 months. Secondary outcomes included mean weight change from baseline at 3 months and 1 year, as well as changes in metabolic profiles such as total cholesterol, triglycerides, and LDL-cholesterol, HDL-cholesterol levels, glycated hemoglobin (HbA1c), fasting blood glucose (FBG), alanine transaminase (ALT), aspartate transaminase (AST), systolic blood pressure (SBP), and diastolic blood pressure (DBP) at all time points. Data on participants’ medications were collected at baseline and during each study visit via survey questions to capture any changes in the type, dose, or frequency of medications. This approach was implemented to monitor and account for potential confounding effects that could influence study outcomes.

### 2.7. Anthropometric and Biochemical Measurements

During the study visits, a calibrated digital weighing scale (Omron HN-289, Omron Healthcare Co. Ltd., Kyoto, Japan) was used to measure the participants’ body weight. An automatic blood pressure monitor (Omron HBP-1300, Omron Healthcare Co. Ltd., Kyoto, Japan) was used for blood pressure measurement. The participants’ blood samples were taken after an overnight fast and analyzed at the National University Hospital Referral Laboratory.

Participants returned to the study site at 3 months, 6 months, and 1 year post-enrollment for body weight measurements and blood tests. Two-day food diaries were collected at all time points to assess energy and nutrients intake. A dietitian performed an analysis of the dietary intake using the localized nutrient analysis platform of the nBuddy app. This platform comprised various food databases, including the Singapore Energy and Nutrient Composition of Food, Malaysian Food Composition, and US Department of Agriculture databases, as well as nutritional information from food packaging and recipes.

### 2.8. Sample Size

The sample size was calculated based on the assumption of a Cohen’s effect size of 0.8 for the difference in weight loss between groups at 6 months. With 80% power at 2-sided 5% level of significance, a minimum of 25 patients in each arm was required. Factoring in a 20% attrition rate, at least 60 subjects would be required (30 per arm).

### 2.9. Statistical Analysis

All statistical analyses were performed using IBM SPSS Statistics (version 29, IBM Corporation, New York, NY, USA). As this study was set out as intention-to-treat, the results were analyzed in the groups to which they were initially randomized, regardless of whether they adhered to the treatment protocol, switched treatments, or dropped out of the study. Differences in continuous variables were assessed using a 2-sample *t*-test, while Chi-square or Fisher’s exact test was used for categorical variables. Linear regression was performed on the change from baseline for each continuous outcome, adjusting for demographics and relevant covariates. Type 1 errors for multiple comparisons were adjusted using the Benjamini–Hochberg procedure with a false discovery rate of 0.20. A comparison of changes from baseline was performed using a paired Student *t*-test. Logistic regression was performed on binary weight loss ≥5% and ≥10%, adjusting for demographics and relevant covariates. Regression analysis between weight loss and reduction in net carbohydrate intake was performed, adjusting for energy intake. Pearson correlation was carried out to determine the relationship between net carbohydrates, energy intake, and weight loss. Statistical significance was set at *p* < 0.05.

## 3. Results

### 3.1. Study Participants

**Figure 1:**
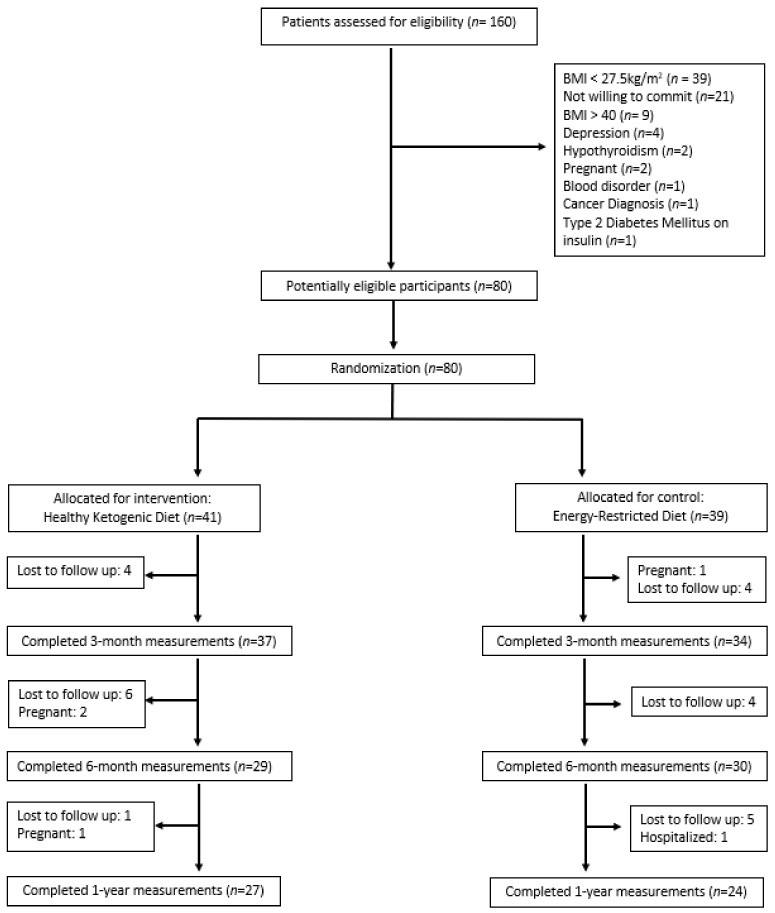
Participant flowchart.

A total of 160 participants were screened, with 80 enrolled and randomized to either the HKD group (*n* = 41) or the ERD group (*n* = 39). The percentage of participants who completed the 3-month, 6-month, and 1-year study was 89%, 74%, and 64%, respectively (Figure 1).

### 3.2. Baseline Characteristics

Table 1 summarizes the participants’ baseline characteristics. Overall, baseline characteristics were comparable between the HKD and ERD groups.

### 3.3. Weight Loss

Table 2 shows changes in weight and metabolic parameters between groups. At 6 months, participants in the HKD group achieved significantly greater reduction in body weight compared with the ERD group (mean weight change ± SD, −7.8 ± 5.2 kg vs. −4.2 ± 5.6 kg; *p* = 0.01). The HKD group was associated with 14.5 times (95% CI 1.6–131.9, *p* = 0.017) and nearly five times (95% CI 1.3–17.8, *p* = 0.017) greater odds of achieving ≥10% weight loss compared to the ERD group at 3 months and 6 months, respectively (Table 3).

### 3.4. Net Carbohydrate Intake and Weight Loss

**Figure 2:**
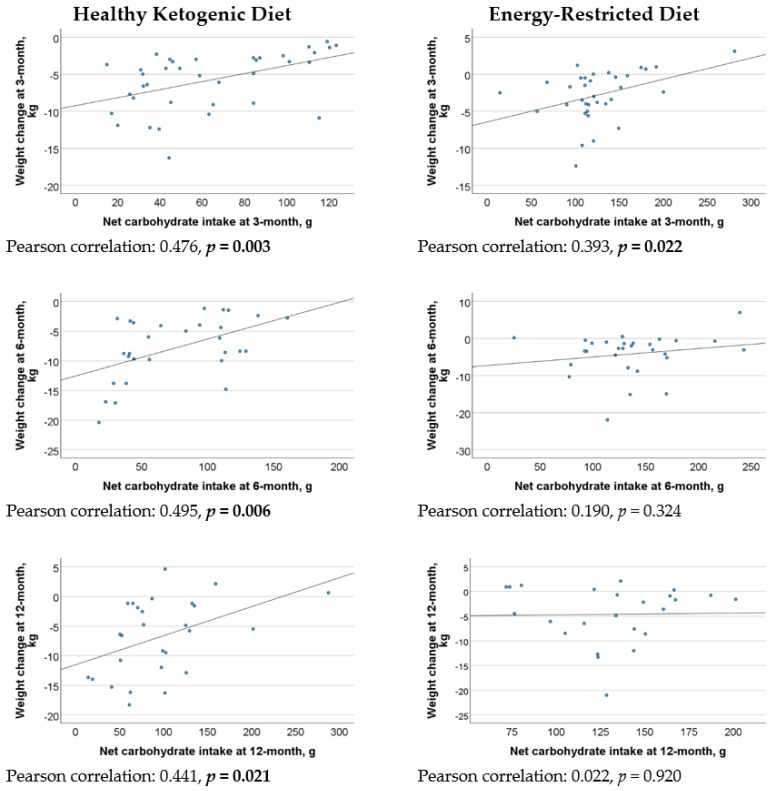
Correlation between net carbohydrate intake and weight loss in the Healthy Ketogenic Diet (HKD) and Energy-Restricted Diet (ERD) groups at 3 months, 6 months, and 1 year.

**Figure 3:**
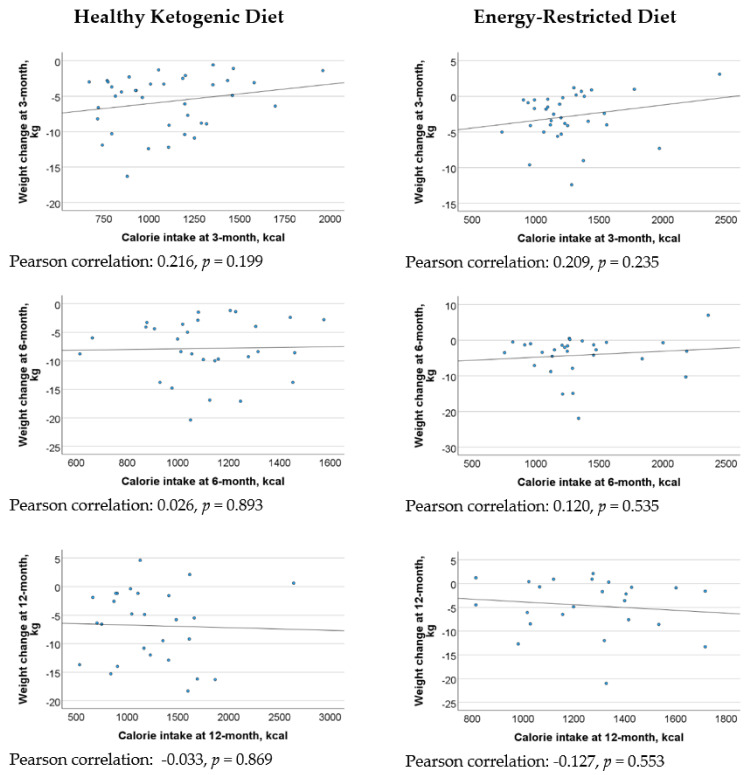
Correlation between energy intake and weight loss in the Healthy Ketogenic Diet (HKD) and Energy-Restricted Diet (ERD) groups at 3 months, 6 months, and 1 year.

Figure 2 revealed a statistically significant moderate positive correlation between net carbohydrate intake and weight loss at 3 months, 6 months, and 1 year in the HKD group. However, there were weak correlations between energy intake and weight loss in both the HKD and ERD groups across the various assessment points (Figure 3). In the regression analysis, every 10 g decrease in net carbohydrate intake resulted in 0.6 kg (95% CI 0.1–1.0, *p* = 0.012), 0.7 kg (95% CI 0.3–1.2, *p* = 0.003), and 1.3 kg (95% CI 0.8–1.8, *p* < 0.001) increase in weight loss in the HKD group at 3 months, 6 months, and 1 year, respectively, after adjusting for energy intake and baseline weight (Table 4).

### 3.5. Cardiometabolic Outcomes

Table 2 shows the changes in the cardiometabolic outcomes between the two intervention groups. Overall, the HKD group demonstrated significant within-group improvement in metabolic outcomes, including HbA1c, fasting blood glucose, blood pressure, liver enzymes, and lipid profiles, at both 3 and 6 months of intervention, as well as 1 year post-enrollment. In contrast, the ERD group showed within-group improvements primarily in HbA1c and fasting blood glucose. Between-group comparisons revealed the HKD group achieving significantly greater reductions in HbA1c, liver enzymes, SBP, total cholesterol, and triglycerides compared with the ERD group.

### 3.6. Medication Changes

In the HKD group, three participants reduced their blood pressure medication dose (one at 3 months and two at 1 year), while one participant required an increased dose at 6 months. For diabetes management, one HKD participant reduced the dose of medication at 3 months, and another adjusted their dosage to a more even distribution throughout the day at the 1-year mark. No participant in the HKD group reported changes to lipid-lowering medication. In contrast, four participants in the ERD group required increased medication doses over the course of the study (two for cholesterol management and two for high blood pressure). Only one participant in the ERD group reported a decrease in blood pressure medication dose.

### 3.7. Dietary Intake

The average energy intake and macronutrient intake distributions were similar between groups at baseline, with a carbohydrate–protein–fat ratio of approximately 44%:18%:38%, respectively. At 6 months, the HKD group consumed 244 kcal (95% CI: 82–406 kcal, *p* = 0.004) less than the ERD group (Table 5). The macronutrient intake distribution in the HKD group shifted to 28% carbohydrate–27% protein–45% fat at 3 months and 33% carbohydrate–24% protein–43% fat at 6 months. Despite the higher proportion of fat intake in the HKD group, participants maintained their total fat (mean: 55 g at 3 months; 53 g at 6 months) and saturated fat intake (mean: 20 g at 3 months; 18 g at 6 months) within the recommended ranges while adhering to energy restriction. In contrast, the macronutrient intake distribution in the ERD group remained consistent throughout the study. Participants following the HKD also consumed significantly lesser carbohydrate, sugar, and sodium compared to both their baseline intake and those on ERD (Table 5). The assessment of micronutrient adequacy was limited, as compliance with the multivitamin-multimineral supplementation recommendation was not tracked. Furthermore, the incomplete micronutrient data on food items and labelling hindered a comprehensive analysis of the diet’s micronutrient profile. Future studies should monitor supplement intake and gather detailed dietary and biochemical data to better assess nutritional status, enhancing understanding of the diet’s impacts, and identify potential deficiencies.

## 4. Discussion

This study is the first to report on the development of the HKD and demonstrate its effectiveness in promoting significant weight loss without increasing LDL-cholesterol levels. Furthermore, the HKD group exhibited greater metabolic improvements compared to the ERD group. At 3 months, participants in the HKD group were 3.5 times more likely to achieve ≥5% weight loss and 14.5 times more likely to achieve ≥10% weight loss compared to those in the ERD group. By 6 months, the likelihood of achieving ≥5% and ≥10% weight loss was 5.6 and 4.9 times higher, respectively, in the HKD group than in the ERD group.

After controlling for the potential confounders of age, gender, and baseline body weight, we found that the HKD group achieved 3.0 kg and 3.6 kg greater mean weight loss than the ERD group at 3 months and 6 months, respectively. Previous meta-analyses have reported a mean difference in weight loss of 2.2–5.6 kg for a conventional ketogenic diet (KD) compared to low-fat diets over 6–12 months [15,28]. In the current study, correlation analysis revealed that a reduction in net carbohydrate intake was significantly associated with increased weight loss, whereas energy intake did not exhibit a similar association. This implies that adherence to the net carbohydrate target is more closely linked to weight loss outcomes. These findings are consistent with those of Li et al., who observed that higher adherence to a KD was associated with more favorable weight loss results [29]. The lack of a significant relationship between energy intake and weight loss may suggest that the two factors may not share a straightforward linear relationship. Instead, multi-faceted variables such as metabolic adaptations, changes to appetite and hormones, and the effects of diet composition play a significant role in influencing weight loss outcomes [30].

The greater weight loss observed in the HKD group could be attributed to several reasons. First, the HKD lowers insulin levels, which may enhance the mobilization of fats as fuel, thereby reducing fat storage leading to weight loss [3,4]. Second, a low carbohydrate intake stabilizes blood glucose levels, which helps regulate hunger and support adherence to caloric limits necessary for weight loss [4,29,31]. Third, significantly reducing carbohydrate intake can induce a state of ketosis, where the body switches to using fat for energy production in the form of ketones, promoting the burning of stored fats and facilitating weight loss. Ketosis also stimulates the release of satiety hormones, including glucagon-like peptide-1 (GLP-1), leptin, cholecystokinin, and peptide YY [4,32,33,34]. Additionally, ketone bodies produced during ketosis may have a satiating effect, further suppressing appetite and enhancing compliance with caloric restrictions, thereby contributing to improved weight loss outcomes [3,32]. The higher satiety might also contribute to the significantly lower energy intake among participants in the HKD group at 6 months, compared with the ERD group.

Traditional KDs have been associated with increased cholesterol levels, raising concerns about cardiovascular health [2,6,28]. However, our study did not observe elevated cholesterol levels at the end of the 6-month intervention period. In fact, participants on the HKD exhibited significantly greater reduction in total cholesterol and triglyceride at one year, along with within-group improvements on these two parameters at all measured time points, and in LDL-cholesterol at 1 year. This favorable outcome may be attributed to the diet’s emphasis on limiting saturated fats and trans fats, while incorporating monounsaturated fats, omega-3 fatty acids, and adequate fiber intake. These findings are consistent with those of Falkenhain et al. and Shai et al., who reported no significant increase in cholesterol levels following a Mediterranean-style KD diet [16,17]. Additionally, the HKD group demonstrated significant improvements in HbA1c, blood pressure, and liver enzymes, supporting existing evidence of HbA1c reductions of 0.2–0.4% with ketogenic diet interventions lasting between three to 12 months [15,16]. The reduction in saturated fat, carbohydrates, and sugar intake has been shown in previous studies to improve insulin sensitivity, which may explain the HKD’s favorable effects on metabolic biomarkers, alongside its role in promoting weight loss [35,36]. Notably, in our study, the improvement in biomarkers was accompanied by a reduction in medication use, suggesting that the HKD may not only enhance cardiometabolic health but also reduce participants’ reliance on pharmaceuticals. Collectively, these findings highlight the HKD as a promising approach for mitigating metabolic disease risk, managing comorbidities, potentially lowering healthcare costs, and improving quality of life.

This study is the first to define and implement the therapeutic requirements of a HKD, demonstrating its effectiveness in achieving weight loss and cardiometabolic benefits in adults with obesity. A key strength is the use of a stratified RCT design, which ensures comparable baseline characteristics between groups, thereby enhancing the robustness of the findings. Furthermore, the application of intention-to-treat analysis which included data from all participants regardless of their adherence to dietary recommendations, minimized type 1 errors, and provided a more accurate assessment of the intervention’s effects. This pragmatic randomized controlled trial evaluated the effectiveness of an HKD intervention in a real-world setting, yielding results that are both generalizable and directly applicable to clinical practice.

There are several limitations in this study. Dietary intake was assessed through self-reported food diaries, which may introduce reporting bias and estimation errors. To mitigate these inaccuracies, research dietitians systematically reviewed and clarified the food diaries with participants, using visual aids to help accurately estimate portion sizes during study visits. Nevertheless, future studies would benefit from combining self-reported and objective data (e.g., blood ketones, urinary ketones) for a more comprehensive analysis of the compliance rate. Conducting the study in a closely monitored clinical setting, with stricter control over dietary adherence and confounding factors, may also yield clearer insights into the physiological mechanisms and true effects of the HKD. Finally, the relatively small sample size and single-center design may limit the generalizability of findings to a broader population, warranting further replication in larger and more diverse cohorts.

## 5. Conclusions

The HKD was more effective than an ERD in promoting weight loss and improving cardiometabolic profiles in individuals with obesity within a short time frame. In contrast to traditional ketogenic diets, the HKD did not cause an increase in LDL-cholesterol. It can be recommended for therapeutic intervention in patients with obesity.

## Figures and Tables

**Table 1:** Baseline characteristics of study participants.

Variable	Healthy Ketogenic Diet (*n* = 41)	Energy-Restricted Diet (*n* = 39)	*p*-Value ^a^
Gender, *n* (%)			
Female	36 (87.8%)	33 (84.6%)	0.679
Male	5 (12.2%)	6 (15.4%)	
Ethnicity, *n* (%)			
Chinese	26 (63.4%)	18 (46.2%)	0.255
Malay	10 (24.4%)	9 (23.1%)	
Indian	3 (7.3%)	7 (17.9%)	
Others	2 (4.9%)	5 (12.8%)	
Age (years)			
Mean	38.4 ± 8.8	39.4 ± 7.6	0.600
Range	22–63	28–62	
Weight, kg	84.2 ± 14.3	83.3 ± 12.2	0.764
Body Mass Index, kg/m^2^	32.4 ± 3.9	31.9 ± 3.4	0.546
HbA1c, %	5.7 ± 0.6	5.5 ± 0.3	0.078
Fasting blood glucose, mmol/L	5.7 ± 1.0	5.4 ± 0.5	0.067
Systolic blood pressure, mmHg	121.7 ± 12.7	122.5 ± 18.5	0.823
Diastolic blood pressure, mmHg	78.0 ± 9.8	78.6 ± 10.5	0.796
Total cholesterol, mmol/L	5.3 ± 0.9	5.0 ± 1.0	0.158
LDL-cholesterol, mmol/L	3.3 ± 0.8	3.2 ± 0.8	0.328
HDL-cholesterol, mmol/L	1.4 ± 0.2	1.3 ± 0.3	0.647
Triglyceride, mmol/L	1.3 ± 0.9	1.1 ± 0.5	0.197
Alanine Transaminase, U/L	34.5 ± 33.1	28.8 ± 23.2	0.377
Aspartate Transaminase, U/L	27.4 ± 16.1	24.6 ± 9.5	0.357
Co-morbidity, *n* (%)			
Hypertension			0.509
No	23 (56.1%)	19 (48.7%)	
Yes	18 (43.9%)	20 (51.3%)	
Hyperlipidemia			0.288
No	4 (9.8%)	7 (17.9%)	
Yes	37 (90.2%)	32 (82.1%)	
Diabetes			0.417 0.117
No	33 (80.5%)	34 (87.2%)	
Yes	8 (19.5%)	5 (12.8%)	
Transaminitis			
No	30 (73.2%)	34 (87.2%)	
Yes	11 (26.8%)	5 (12.8%)	
Nutrient intake			
Energy, kcal	1858 ± 400	1786 ± 460	0.459
Protein, g	83.9 ± 25.2	75.6 ± 16.7	0.086
Total fat, g	81.0 ± 23.0	75.2 ± 22.6	0.259
Saturated fat, g	29.7 ± 10.3	29.1 ± 8.9	0.766
Carbohydrate, g	202.4 ± 56.3	202.0 ± 58.6	0.979
Net Carbohydrate, g	185.1 ± 55.0	186.0 ± 56.9	0.940
Sugar, g	53.1 ± 29.1	48.9 ± 22.3	0.468
Fiber, g	16.9 ± 5.9	16.3 ± 6.0	0.647
Sodium, mg	3402 ± 1027	3190 ± 1015	0.355

Data expressed as mean ± SD for continuous variables; absolute numbers (percentages) for categorical variables. HbA1c, Glycated hemoglobin; LDL, Low-density Lipoprotein; HDL, High-density Lipoprotein. ^a^ Chi-square, Fisher exact, independent samples *t*-test as appropriate.

**Table 2:** Primary and secondary outcomes at 3 months, 6 months, and 1 year after enrollment.

Outcomes	*n*	Mean Change from Baseline		Between-Group Differences					
				Unadjusted			Adjusted ^a^		
		Healthy Ketogenic Diet (*n* = 41)	Energy- Restricted Diet (*n* = 39)	Mean Difference (95% CI)	*p*-Value	Cohen d	Mean Difference (95% CI)	*p*-Value	Cohen d
∆ Weight, kg									
3 months	72	−5.8 ± 3.9 *	−2.8 ± 3.3 *	−3.0 (−4.7–−1.3)	**0.001**	0.83	−3.0 (−4.6–−1.4)	**<0.001**	0.44
6 months	59	−7.8 ± 5.2 *	−4.2 ± 5.6 *	−3.7 (−6.5–−0.9)	**0.012**	0.67	−3.6 (−6.4–−0.9)	**0.010**	0.35
12 months	51	−6.9 ± 6.4 *	−4.6 ± 5.8 *	−2.2 (−5.6–1.2)	0.202	0.38	−1.5 (−4.8–1.7)	0.353	0.13
∆ Weight, %									
3 months	72	−6.8 ± 4.2	−3.3 ± 3.6	−3.5 (−5.4–−1.7)	**<0.001**	0.89	−3.7 (−5.6–−1.9) ^b^	**<0.001 ^b^**	0.49 ^b^
6 months	59	−9.3 ± 5.9	−4.9 ± 5.8	−4.4 (−7.5–−1.4)	**0.005**	0.75	−4.6 (−7.6–−1.5) ^b^	**0.004 ^b^**	0.39 ^b^
12 months	51	−7.9 ± 7.7	−5.4 ± 6.2	−2.6 (−6.5–1.4)	0.199	0.36	−2.7 (−6.6–1.3) ^b^	0.177 ^b^	0.19 ^b^
∆ BMI, kg/m^2^									
3 months	72	−2.2 ± 1.4 *	−1.0 ± 1.2 *	−1.2 (−1.8–−0.6)	**<0.001**	0.92	−1.2 (−1.8–−0.6)	**<0.001**	0.48
6 months	59	−3.0 ± 2.0 *	−1.5 ± 1.9 *	−1.5 (−2.5–−0.5)	**0.004**	0.77	−1.6 (−2.6–−0.5)	**0.003**	0.40
12 months	51	−2.6 ± 2.5 *	−1.7 ± 2.0 *	−0.9 (−2.2–0.3)	0.142	0.40	−1.0 (−2.2–0.3)	0.128	0.22
∆ ALT, U/L									
3 months	71	−14.4 ± 27.8 *	−4.9 ± 15.9	−9.5 (−20.3–1.4)	0.086	0.42	−3.6 (−8.0–0.9)	0.113	0.19
6 months	59	−19.0 ± 32.5 *	−5.7 ± 23.4	−13.3 (−28.0–1.4)	0.076	0.47	−4.0 (−8.0–0.0)	0.051	0.26
12 months	51	−19.2 ± 38.2 *	−0.9 ± 23.8	−18.3 (−36.4–−0.1)	**0.049**	0.58	−11.1 (−21.9–−0.2)	**0.045**	0.29
∆ AST, U/L									
3 months	71	−6.7 ± 14.5 *	−1.4 ± 8.7	−5.3 (−11.0–0.5)	0.071	0.44	−2.3 (−5.1–0.6)	0.124	0.19
6 months	59	−7.6 ± 15.5 *	0.6 ± 11.5	−8.2 (−15.3–−1.1)	**0.024**	0.60	−4.1 (−7.1–−1.0)	**0.010**	0.35
12 months	51	−8.3 ± 18.2 *	1.8 ± 11.7	−10.1 (−18.9–−1.4)	**0.024**	0.66	−5.5 (−10.4–−0.6)	**0.028**	0.32
∆ HbA1c, %									
3 months	71	−0.3 ± 0.3 *	−0.1 ± 0.2 *	−0.2 (−0.3–−0.1)	**0.005**	0.78	−0.1 (−0.2–0.00)	0.060	0.24
6 months	59	−0.3 ± 0.3 *	−0.1 ± 0.2	−0.2 (−0.3–−0.1)	**0.002**	0.78	−0.2 (−0.3–−0.0)	**0.008**	0.37
12 months	51	−0.3 ± 0.3 *	−0.1 ± 0.2 *	−0.2 (−0.3–0.0)	**0.037**	0.78	−0.1 (−0.2–0.0)	0.145	0.21
∆ Fasting Blood Glucose, mmol/L									
3 months	71	−0.4 ± 0.5 *	−0.2 ± 0.6 *	−0.1 (−0.4–0.1)	0.277	0.36	0.0 (−0.2–0.2)	0.972	0.00
6 months	59	−0.4 ± 0.5 *	−0.2 ± 0.5 *	−0.2 (−0.4–0.1)	0.172	0.40	−0.1 (−0.4–0.2)	0.415	0.10
12 months	51	−0.3 ± 0.5 *	−0.2 ± 0.5 *	−0.1 (−0.4–0.2)	0.421	0.20	−0.0 (−0.3–0.3)	0.924	0.01
∆ Systolic blood pressure, mmHg									
3 months	71	−7.1 ± 7.4 *	−4.1 ± 14.5	−3.0 (−8.3–2.4)	0.275	0.26	−3.5 (−7.9–0.9)	0.121	0.19
6 months	59	−7.7 ± 8.9 *	−2.6 ± 12.2	−5.1 (−10.7–0.5)	0.071	0.48	−6.4 (−10.7–−2.0)	**0.005**	0.38
12 months	51	−4.2 ± 10.7	−5.7 ± 9.5 *	1.5 (−4.2–7.2)	0.605	0.15	0.4 (−4.7–5.5)	0.887	0.02
∆ Diastolic blood pressure, mmHg									
3 months	71	−3.1 ± 7.0 *	−5.0 ± 9.9 *	1.9 (−2.2–5.9)	0.359	0.22	1.5 (−2.2–5.2)	0.424	0.10
6 months	59	−3.7 ± 6.5 *	−2.4 ± 7.7	−1.4 (−5.1–2.4)	0.468	0.18	−1.6 (−5.0–1.9)	0.359	0.12
12 months	51	−2.1 ± 8.8	−2.6 ± 9.2	0.5 (−4.6–5.6)	0.841	0.06	0.3 (−4.6–5.1)	0.917	0.01
∆ Total cholesterol, mmol/L									
3 months	71	−0.42 ± 0.75 *	−0.16 ± 0.55	−0.26 (−0.57–0.05)	0.098	0.40	−0.13 (−0.39–0.13)	0.331	0.12
6 months	59	−0.29 ± 0.75 *	−0.08 ± 0.67	−0.21 (−0.58–0.17)	0.273	0.30	−0.10 (−0.43–0.23)	0.551	0.08
12 months	51	−0.44 ± 0.71 *	0.06 ± 0.87	−0.50 (−0.95–−0.06)	**0.027**	0.63	−0.47 (−0.91–−0.03)	**0.036**	0.30
∆ HDL-cholesterol, mmol/L									
3 months	71	−0.04 ± 0.24	−0.01 ± 0.17	−0.02 (−0.12–0.07)	0.626	0.14	−0.01 (−0.11–0.08)	0.760	0.03
6 months	59	0.05 ± 0.19	0.01 ± 0.20	0.04 (−0.06–0.15)	0.404	0.21	0.04 (−0.06–0.14)	0.406	0.11
12 months	51	0.09 ± 0.19 *	0.01 ± 0.21	0.08 (−0.03–0.19)	0.161	0.40	0.08 (−0.03–0.18)	0.161	0.21
∆ Triglycerides, mmol/L									
3 months	71	−0.44 ± 0.68 *	−0.06 ± 0.39	−0.38 (−0.65–−0.12)	**0.005**	0.69	−0.20 (−0.34–−0.06)	**0.006**	0.33
6 months	59	−0.49 ± 0.80 *	−0.13 ± 0.57	−0.36 (−0.72–0.00)	0.051	0.52	−0.17 (−0.42–0.08)	0.181	0.18
12 months	51	−0.57 ± 0.88 *	−0.04 ± 0.66	−0.53 (−0.98–−0.09)	**0.020**	0.68	−0.40 (−0.78–−0.03)	**0.036**	0.30
∆ LDL-cholesterol, mmol/L									
3 months	71	−0.19 ± 0.69	−0.11 ± 0.39	−0.08 (−0.35–0.19)	0.554	0.14	−0.01 (−0.24–0.22)	0.924	0.01
6 months	59	−0.12 ± 0.60	−0.04 ± 0.56	−0.08 (−0.38–0.23)	0.620	0.14	0.00 (−0.27–0.26)	0.970	0.00
12 months	51	−0.27 ± 0.58 *	0.07 ± 0.76	−0.35 (−0.72–0.03)	0.070	0.50	−0.33 (−0.69–0.03)	0.073	0.26

Data expressed as mean ± SD. BMI, Body Mass Index; ALT, Alanine Transaminase; AST, Aspartate Transaminase, HbA1c, Glycated Hemoglobin; HDL, High-density Lipoprotein; LDL, Low-density Lipoprotein. ^a^ Adjusted for gender, age, and baseline value of the outcome. ^b^ Adjusted for gender and age. * Significant within-group changes *p*-values after Benjamini–Hochberg correction with false discovery rate at 0.20 and *n* = 72. In bold: Significant between-group differences *p*-Values after Benjamini–Hochberg correction with false discovery rate at 0.20 and *n* = 78.

**Table 3:** Odds ratios of the Healthy Ketogenic Diet (HKD) group achieving weight loss at 3 months, 6 months, and 1 year in comparison to the Energy-Restricted Diet (ERD) group.

			**Unadjusted**		**Adjusted ^a^**	
	**Weight Loss < 5%**	**Weight Loss ≥ 5%**	**OR (95% CI)**	***p*-Value**	**OR (95% CI)**	***p*-Value**
3 months						
HKD	15 (40.5%)	22 (59.5%)	3.2 (1.2–8.4)	**0.019**	3.5 (1.3–9.4)	**0.014**
ERD	24 (68.6%)	11 (31.4%)	1.0		1.0	
6 months						
HKD	8 (27.6%)	21 (72.4%)	5.3 (1.7–16.0)	**0.004**	5.6 (1.8–17.6)	**0.003**
ERD	20 (66.7%)	10 (33.3%)	1.0		1.0	
12 months						
HKD	11 (40.7%)	16 (59.3%)	1.5 (0.5–4.4)	0.508	1.5 (0.5–4.7)	0.474
ERD	12 (50.0%)	12 (50.0%)	1.0		1.0	
			**Unadjusted**		**Adjusted ^a^**	
	**Weight loss < 10%**	**Weight loss ≥ 10%**	**OR (95% CI)**	***p*-Value**	**OR (95% CI)**	***p*-Value**
3 months						
HKD	28 (75.7%)	9 (24.3%)	10.9 (1.3–91.6)	**0.027**	14.5 (1.6–131.9)	**0.017**
ERD	34 (97.1%)	1 (2.9%)	1.0		1.0	
6 months						
HKD	16 (55.2%)	13 (44.8%)	4.1 (1.2–13.6)	**0.023**	4.9 (1.3–17.8)	**0.017**
ERD	25 (83.3%)	5 (16.7%)	1.0		1.0	
12 months						
HKD	16 (59.3%)	11 (40.7%)	2.6 (0.7–9.1)	0.132	3.2 (0.8–12.3)	0.099
ERD	19 (79.2%)	5 (20.8%)	1.0		1.0	

^a^ Adjusted for gender and age. In bold: Significant *p*-Values after Benjamini–Hochberg correction with false discovery rate at 0.20 and *n* = 12.

**Table 4:** Regression analysis of weight loss for every 10-gram reduction in net carbohydrate intake in the Healthy Ketogenic Diet (HKD) group.

		Unadjusted		Adjusted ^a^	
Time Point	*n*	Weight Loss in kg for Every 10 g Decrease in Net Carbohydrate, B (95% CI)	*p*-Value	Weight Loss in kg for Every 10 g Decrease in Net Carbohydrate, B (95% CI)	*p*-Value
3 months	37	0.5 (0.2–0.9)	**0.003**	0.6 (0.1–1.0)	**0.012**
6 months	29	0.6 (0.2–1.1)	**0.006**	0.7 (0.3–1.2)	**0.003**
12 months	27	0.5 (0.1–0.9)	**0.021**	1.3 (0.8–1.8)	**<0.001**

^a^ Adjusted for energy intake and baseline weight. In bold: Significant *p*-values after Benjamini–Hochberg correction with false discovery rate at 0.20 and *n* = 6.

**Table 5:** Nutrition outcomes at 3 months, 6 months and 1 year after enrollment.

Outcomes	*n*	Mean Change from Baseline		Between-Group Differences					
				Unadjusted			Adjusted ^a^		
		Healthy Ketogenic Diet (*n* = 41)	Energy- Restricted Diet (*n* = 39)	Mean Difference (95% CI)	*p*-Value	Cohen d	Mean Difference (95% CI)	*p*-Value	Cohen d
∆ Energy, kcal									
3 months	72	−736 ± 445 *	−571 ± 465 *	−166 (−380–48)	0.127	0.36	−121 (−270–28)	0.110	0.19
6 months	58	−704 ± 372 *	−423 ± 510 *	−281 (−516–−46)	**0.020**	0.63	−244 (−406–−82)	**0.004**	0.40
12 months	51	−592 ± 469 *	−499 ± 518 *	−92 (−370–185)	0.507	0.19	−43 (−255–169)	0.687	0.06
∆ Protein, g									
3 months	71	−11.5 ± 27.6 *	−13.7 ± 20.1 *	2.1 (−9.4–13.7)	0.713	0.09	8.0 (−1.7–17.8)	0.105	0.19
6 months	58	−17.1 ± 31.2 *	−7.5 ± 33.9	−9.6 (−26.7–7.5)	0.265	0.29	−2.2 (−17.4–12.9)	0.769	0.04
12 months	51	−15.7 ± 27.1 *	−13.3 ± 21.6 *	−2.4 (−16.3–11.5)	0.732	0.10	4.9 (−6.8–16.7)	0.404	0.12
∆ Total fat, g									
3 months	71	−26.2 ± 24.2 *	−26.2 ± 23.1 *	0.003 (−11.216–11.222)	1.000	0.0001	4.8 (−3.5–13.2)	0.253	0.14
6 months	58	−25.3 ± 22.1 *	−22.2 ± 27.3 *	−3.1 (−16.2–9.9)	0.634	0.12	0.7 (−9.8–11.2)	0.891	0.02
12 months	51	−22.8 ± 28.9 *	−26.7 ± 28.8 *	3.9 (−12.4–20.1)	0.635	0.14	10.0 (−1.9–22.0)	0.098	0.24
∆ Saturated fat, g									
3 months	71	−9.8 ± 12.8 *	−10.4 ± 9.2 *	0.6 (−4.7–5.9)	0.830	0.05	1.1 (−2.8–5.0)	0.582	0.07
6 months	58	−11.9 ± 10.8 *	−9.2 ± 11.1 *	−2.7 (−8.5–3.0)	0.349	0.25	−1.3 (−5.2–2.5)	0.494	0.09
12 months	51	−10.0 ± 13.6 *	−10.5 ± 9.4 *	0.5 (−6.2–7.1)	0.890	0.04	2.5 (−2.1–7.1)	0.275	0.15
∆ Carbohydrate, g									
3 months	71	−119.0 ± 64.6 *	−58.8 ± 62.8 *	−60.3 (−90.5–−30.1)	**<0.001**	0.94	−67.7 (−85.9–−49.5)	**<0.001**	0.88
6 months	57	−106.0 ± 64.3 *	−48.0 ± 68.2 *	−58.0 (−93.2–−22.8)	**0.002**	0.88	−61.7 (−84.7–−38.8)	**<0.001**	0.71
12 months	51	−85.0 ± 80.1 *	−58.3 ± 64.2 *	−26.7 (−67.9–14.4)	0.198	0.37	−29.8 (−58.5–−1.1)	**0.042**	0.29
∆Net Carbohydrate, g									
3 months	72	−113.8 ± 62.7 *	−69.8 ± 70.8 *	−43.9 (−75.4–−12.5)	**0.007**	0.66	−54.1 (−74.4–−33.7)	**<0.001**	0.62
6 months	58	−106.3 ± 65.9 *	−49.2 ± 66.4 *	−57.1 (−91.9–−22.3)	**0.002**	0.86	−61.3 (−85.2–−37.3)	**<0.001**	0.67
12 months	51	−86.3 ± 76.4 *	−54.2 ± 62.8 *	−32.1 (−71.8–7.5)	0.110	0.46	−35.8 (−63.3–−8.3)	**0.012**	0.37
∆ Sugar, g									
3 months	71	−31.8 ± 29.3 *	−14.9 ± 27.3 *	−16.8 (−30.3–−3.4)	**0.015**	0.60	−14.6 (−20.9–−8.3)	**<0.001**	0.55
6 months	58	−27.0 ± 29.4 *	−12.9 ± 31.4 *	−14.1 (−30.1–1.9)	0.083	0.46	−11.8 (−20.1–−3.5)	**0.006**	0.37
12 months	51	−19.0 ± 30.1 *	−16.5 ± 31.6 *	−2.6 (−19.9–14.8)	0.767	0.08	−2.2 (−14.7–10.4)	0.730	0.05
∆ Fiber, g									
3 montsh	71	−3.5 ± 7.1 *	−3.6 ± 6.3 *	0.1 (−3.1–3.3)	0.963	0.01	−0.1 (−2.6–2.3)	0.909	0.01
6 months	58	−1.6 ± 6.8	−3.7 ± 7.6 *	2.1 (−1.7–5.9)	0.271	0.29	2.0 (−0.7–4.7)	0.148	0.19
12 months	51	−2.0 ± 6.8	−4.4 ± 6.3 *	2.4 (−1.3–6.1)	0.201	0.37	3.4 (0.4–6.3)	**0.028**	0.32
∆ Sodium, mg									
3 months	71	−1320 ± 1192 *	−757 ± 1116 *	−563 (−1111–−15)	**0.044**	0.49	−458 (−830–−86)	**0.017**	0.29
6 months	58	−1156 ± 957 *	−705 ± 1145 *	−451 (−1006–104)	0.109	0.43	−461 (−860–−62)	**0.024**	0.30
12 months	49	−921 ± 1055 *	−555 ± 1103 *	−366 (−980–248)	0.236	0.34	−361 (−862–140)	0.153	0.21

Data expressed as mean ± SD. ^a^ Adjusted for gender, age and baseline value of the outcome. * Significant within-group changes *p* values after Benjamini–Hochberg correction with false discovery rate at 0.20 and *n* = 54. In bold: Significant between-group differences *p*-Values after Benjamini–Hochberg correction with false discovery rate at 0.20 and *n* = 54.

## Data Availability

The data presented in this study are available on request from the corresponding author.

## References

[1] Elsahoryi N.A., Alkurd R.A., Subih H., Musharbash R. (2023). Effect of Low-Calorie Ketogenic vs. Low-Carbohydrate Diets on Body Composition and Other Biomarkers of Overweight/Obese Women: An 8 Weeks Randomised Controlled Trial. Obes. Med..

[2] Bueno N.B., de Melo I.S., de Oliveira S.L., da Rocha Ataide T. (2013). Very-Low-Carbohydrate Ketogenic Diet V. Low-Fat Diet for Long-Term Weight Loss: A Meta-Analysis of Randomised Controlled Trials. Br. J. Nutr..

[3] Kirkpatrick C.F., Bolick J.P., Kris-Etherton P.M., Sikand G., Aspry K.E., Soffer D.E., Willard K.E., Maki K.C. (2019). Review of Current Evidence and Clinical Recommendations on the Effects of Low-Carbohydrate and Very-Low-Carbohydrate (Including Ketogenic) Diets for the Management of Body Weight and Other Cardiometabolic Risk Factors: A Scientific Statement from the National Lipid Association Nutrition and Lifestyle Task Force. J. Clin. Lipidol..

[4] Mohorko N., Cernelic-Bizjak M., Poklar-Vatovec T., Grom G., Kenig S., Petelin A., Jenko-Praznikar Z. (2019). Weight Loss, Improved Physical Performance, Cognitive Function, Eating Behavior, and Metabolic Profile in a 12-Week Ketogenic Diet in Obese Adults. Nutr. Res..

[5] Hall K.D., Guo J., Courville A.B., Boring J., Brychta R., Chen K.Y., Darcey V., Forde C.G., Gharib A.M., Gallagher I. (2021). Effect of a Plant-Based, Low-Fat Diet Versus an Animal-Based, Ketogenic Diet on Ad Libitum Energy Intake. Nat. Med..

[6] Gjuladin-Hellon T., Davies I.G., Penson P., Amiri Baghbadorani R. (2019). Effects of Carbohydrate-Restricted Diets on Low-Density Lipoprotein Cholesterol Levels in Overweight and Obese Adults: A Systematic Review and Meta-Analysis. Nutr. Rev..

[7] Buren J., Ericsson M., Damasceno N.R.T., Sjodin A. (2021). A Ketogenic Low-Carbohydrate High-Fat Diet Increases LDL Cholesterol in Healthy, Young, Normal-Weight Women: A Randomized Controlled Feeding Trial. Nutrients.

[8] Batch J.T., Lamsal S.P., Adkins M., Sultan S., Ramirez M.N. (2020). Advantages and Disadvantages of the Ketogenic Diet: A Review Article. Cureus.

[9] Muscogiuri G., El Ghoch M., Colao A., Hassapidou M., Yumuk V., Busetto L. (2021). European Guidelines for Obesity Management in Adults with a Very Low-Calorie Ketogenic Diet: A Systematic Review and Meta-Analysis. Obes. Facts.

[10] Barber T.M., Kabisch S., Pfeiffer A.F.H., Weickert M.O. (2020). The Health Benefits of Dietary Fibre. Nutrients.

[11] Reynolds A.N., Akerman A., Kumar S., Diep Pham H.T., Coffey S., Mann J. (2022). Dietary Fibre in Hypertension and Cardiovascular Disease Management: Systematic Review and Meta-Analyses. BMC Med..

[12] Holscher H.D. (2017). Dietary Fiber and Prebiotics and the Gastrointestinal Microbiota. Gut Microbes.

[13] Caleyachetty R., Barber T.M., Mohammed N.I., Cappuccio F.P., Hardy R., Mathur R., Banerjee A., Gill P. (2021). Ethnicity-Specific BMI Cutoffs for Obesity Based on Type 2 Diabetes Risk in England: A Population-Based Cohort Study. Lancet Diabetes Endocrinol..

[14] Schofield W.N. (1985). Predicting Basal Metabolic Rate, New Standards and Review of Previous Work. Hum. Nutr. Clin. Nutr..

[15] Zhou C., Wang M., Liang J., He G., Chen N. (2022). Ketogenic Diet Benefits to Weight Loss, Glycemic Control, and Lipid Profiles in Overweight Patients with Type 2 Diabetes Mellitus: A Meta-Analysis of Randomized Controlled Trails. Int. J. Environ. Res. Public Health.

[16] Falkenhain K., Locke S.R., Lowe D.A., Reitsma N.J., Lee T., Singer J., Weiss E.J., Little J.P. (2021). Keyto App and Device Versus Ww App on Weight Loss and Metabolic Risk in Adults with Overweight or Obesity: A Randomized Trial. Obesity.

[17] Shai I., Schwarzfuchs D., Henkin Y., Shahar D.R., Witkow S., Greenberg I., Golan R., Fraser D., Bolotin A., Vardi H. (2008). Weight Loss with a Low-Carbohydrate, Mediterranean, or Low-Fat Diet. N. Engl. J. Med..

[18] Bodnaruc A.M., Prud’homme D., Blanchet R., Giroux I. (2016). Nutritional Modulation of Endogenous Glucagon-Like Peptide-1 Secretion: A Review. Nutr. Metab..

[19] Victoria State Government Dietary Fibre. https://www.betterhealth.vic.gov.au/health/healthyliving/fibre-in-food.

[20] Health Promotion Board Singapore High Fibre for a Fit and Fabulous You. https://www.healthhub.sg/live-healthy/more-fibre-for-a-fit-and-fabulous-you.

[21] Queensland Health (2022). Estimating Energy. Protein and Fluid Requirements for Adult Clinical Conditions.

[22] NHS Water, Drinks and Hydration. https://www.nhs.uk/live-well/eat-well/food-guidelines-and-food-labels/water-drinks-nutrition/.

[23] Crosby L., Davis B., Joshi S., Jardine M., Paul J., Neola M., Barnard N.D. (2021). Ketogenic Diets and Chronic Disease: Weighing the Benefits against the Risks. Front. Nutr..

[24] Schutz Y. (2011). Protein Turnover, Ureagenesis and Gluconeogenesis. Int. J. Vitam. Nutr. Res..

[25] Lim S.L., Ong K.W., Johal J., Han C.Y., Yap Q.V., Chan Y.H., Zhang Z.P., Chandra C.C., Thiagarajah A.G., Khoo C.M. (2021). A Smartphone App-Based Lifestyle Change Program for Prediabetes (D’lite Study) in a Multiethnic Asian Population: A Randomized Controlled Trial. Front. Nutr..

[26] Lim S.L., Ong K.W., Johal J., Han C.Y., Yap Q.V., Chan Y.H., Chooi Y.C., Zhang Z.P., Chandra C.C., Thiagarajah A.G. (2021). Effect of a Smartphone App on Weight Change and Metabolic Outcomes in Asian Adults with Type 2 Diabetes: A Randomized Clinical Trial. JAMA Netw. Open.

[27] Lim S.L., Johal J., Ong K.W., Han C.Y., Chan Y.H., Lee Y.M., Loo W.M. (2020). Lifestyle Intervention Enabled by Mobile Technology on Weight Loss in Patients with Nonalcoholic Fatty Liver Disease: Randomized Controlled Trial. JMIR Mhealth Uhealth.

[28] Mansoor N., Vinknes K.J., Veierod M.B., Retterstol K. (2016). Effects of Low-Carbohydrate Diets V. Low-Fat Diets on Body Weight and Cardiovascular Risk Factors: A Meta-Analysis of Randomised Controlled Trials. Br. J. Nutr..

[29] Li S., Du Y., Meireles C., Sharma K., Qi L., Castillo A., Wang J. (2023). Adherence to Ketogenic Diet in Lifestyle Interventions in Adults with Overweight or Obesity and Type 2 Diabetes: A Scoping Review. Nutr. Diabetes.

[30] Benton D., Young H.A. (2017). Reducing Calorie Intake May Not Help You Lose Body Weight. Perspect. Psychol. Sci..

[31] Cheung L.T.F., Ko G.T.C., Chow F.C.C., Kong A.P.S. (2018). Association between Hedonic Hunger and Glycemic Control in Non-Obese and Obese Patients with Type 2 Diabetes. J. Diabetes Investig..

[32] Sumithran P., Prendergast L.A., Delbridge E., Purcell K., Shulkes A., Kriketos A., Proietto J. (2013). Ketosis and Appetite-Mediating Nutrients and Hormones after Weight Loss. Eur. J. Clin. Nutr..

[33] Nymo S., Coutinho S.R., Jorgensen J., Rehfeld J.F., Truby H., Kulseng B., Martins C. (2017). Timeline of Changes in Appetite During Weight Loss with a Ketogenic Diet. Int. J. Obes..

[34] Martins C., Nymo S., Truby H., Rehfeld J.F., Hunter G.R., Gower B.A. (2020). Association between Ketosis and Changes in Appetite Markers with Weight Loss Following a Very Low-Energy Diet. Obesity.

[35] Vessby B., Uusitupa M., Hermansen K., Riccardi G., Rivellese A.A., Tapsell L.C., Nälsen C., Berglund L., Louheranta A., Rasmussen B.M. (2001). Substituting Dietary Saturated for Monounsaturated Fat Impairs Insulin Sensitivity in Healthy Men and Women: The Kanwu Study. Diabetologia.

[36] Foley P.J. (2021). Effect of Low Carbohydrate Diets on Insulin Resistance and the Metabolic Syndrome. Curr. Opin. Endocrinol. Diabetes.

